# Study on vapor–liquid equilibrium and microscopic properties of DL-proline, L-glutamic acid, and L-serine aqueous solutions

**DOI:** 10.3389/fchem.2022.1005291

**Published:** 2022-10-13

**Authors:** Weiping Liu, Yu Zhou, Xianzhen Xu

**Affiliations:** Shandong Sino-Japanese Center for Collaborative Research of Carbon Nanomaterials, College of Chemistry and Chemical Engineering, Instrumental Analysis Center of Qingdao University, Qingdao University, Qingdao, China

**Keywords:** VLE, amino acid, modeling, FTIR, phase equilibria

## Abstract

The vapor–liquid equilibrium (VLE) in aqueous amino acid solutions is essential in chemical production, such as purification, isolation, or crystallization of amino acid intermediates. In this work, VLE of DL-proline, L-glutamic acid, and L-serine aqueous solutions was measured at pressures ranging from 4.82 to 102.58 kPa. The developed model was successfully applied to correlate experimental data with temperatures in the range of 298.15–382.75 K. Model parameters (*h*, $\tau_{w,i}^{\left(0\right)}$ , $\tau_{w,i}^{\left(1\right)}$ , $\tau_{i,w}^{\left(0\right)}$ , and $\tau_{i,w}^{\left(1\right)}$ ) were given. Moreover, the amino acid aqueous solution was investigated by Fourier transform infrared spectroscopy (FTIR). By analyzing infrared spectra, the strength of intermolecular interactions was obtained, and the structure–activity relationship between the microscopic interactions and the VLE was established.

## 1 Introduction

Amino acids are necessary for human life (Yu et al., 2021; Rondanelli et al., 2016) and play a very important role in affecting the digestion, absorption, and metabolism of proteins. In addition, amino acids are also intermediates and reactants of many clinical drugs (Smith et al., 2020; Bakpa et al., 2021) and are widely used in the synthesis of catalysts—asymmetric chiral catalysis and chemical fertilizers (Fernandez et al., 2022; Nematzadeh et al., 2020). In industry, amino acid salt solutions were often used as an excellent substance for absorbing carbon dioxide because amino acids are green chemicals and are harmless to the environment(Chen et al., 2015). In addition, amino acid salts are not easily volatile (Aronu et al., 2010). The energy required in the process of absorbing carbon dioxide is low (Masoumi et al., 2016; Zarei et al., 2020).

It is very significant to study the properties of amino acids, while solubility is an important physicochemical property that determines the phase equilibrium (Do et al., 2021; Sadeghi et al., 2021). Phase equilibrium has been widely used as the theoretical basis for the separation and purification of inorganic salts, organic molecules, and biological macromolecules in the chemical industry (Vartak et al., 2018; Zhu et al., 2020; Huang et al., 2021). In recent years, research on phase equilibrium has mainly focused on single electrolyte solution systems and mixed electrolyte solutions systems (Huang et al., 2021; Xu et al., 2019; Cui et al., 2021). The relationship between temperature, solubility, and saturated vapor pressure of a single solute in different solvents was studied (Xu et al., 2018). For the phase equilibria of inorganic salts, most authors mainly focused on the study of sodium chloride, calcium chloride, and potassium bromide, to study their phase changes within a certain temperature range and a fixed temperature (Xu et al., 2018; Zhang et al., 2020). Furthermore, the vapor–liquid equilibrium (VLE) of some amino acid salt solutions has been studied and applied to industrial production and living environments (Masoumi et al., 2016). The VLE in 3-(methylamino)propylamine/sarcosine aqueous solution was measured, and the carbon dioxide absorption equilibrium was experimented by Aronu et al. (Masoumi et al., 2016). Zafarani-Moattar et al. (2016) determined phase equilibria on glycine + choline chloride ionic liquid solutions. Haghtalab et al. performed a high-pressure VLE determination of the solubility of (diisopropylamine + L-lysine) and (diisopropylamine + piperazine + L-lysine) in aqueous solvents (Ali et al., 2021). In conclusion, their research was mainly reflected in the macroscopic thermodynamic VLE. In addition to this, numerous authors have studied other solutions to predict or determine phase equilibria. Sadowski et al. used the advanced ePC-SAFT thermodynamic equation of state to predict pH in a multiphase multicomponent system. Proton activity is used to predict pH in multiphase systems, and the developed framework considers reaction and phase equilibria (Gabriele et al., 2022). Macedo et al. used the PDH (Pitzer–Debye–Hückel equation) + unique model to determine the degree of dissociation of ionic liquids in water (Eugenia et al., 2022). However, to the best of our knowledge, studies on amino acid phase equilibria and their microscopic mechanism of action have been sparse until now.

**FIGURE 1:**
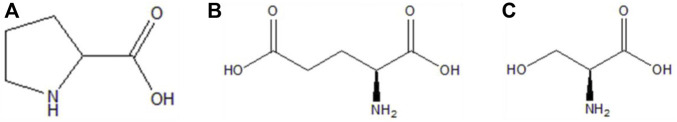
**(A)** Chemical structure of DL-proline. **(B)** Chemical structure of L-glutamic acid. **(C)** Chemical structure of L-serine.

Therefore, in this work, VLE data for three amino acids (DL-proline, L-glutamic acid, and L-serine) were measured. The chemical structures of three different amino acids are shown in Figure 1. In addition, the infrared spectra of three different amino acid aqueous solutions and the infrared spectra of each amino acid at different concentrations were discussed in this work. VLE was mainly used to analyze the thermodynamic differences of different amino acids from a macro-perspective. In contrast, infrared spectra generally analyze the reasons for the differences in the solubility of amino acid aqueous solutions from a microscopic perspective. By observing the wavenumber positions of the characteristic absorption peaks of amino acid aqueous solutions in the infrared spectra, the interaction between molecules in the amino acid aqueous solution, that is, the strength of hydrogen bonds could be judged. In addition, the experimental data of VLE were correlated using the NRTL-X model (Xu et al., 2019). In addition, the five parameters of the model equation were obtained, and at the same time, the correlation analysis was carried out on the binary vapor–liquid equilibrium data at different temperatures and concentrations (Xu et al., 2014).

## 2 Experimental process

### 2.1 Materials

Chemicals used include L-serine (CAS: 56-45-1), DL-proline (CAS: 609-36-9), and L-glutamic acid (CAS: 56-86-0). The solvent used in the experiment is water (conductivity: 18.2 MΩ·cm). Table 1 reports more details about the chemicals used in this work. Tables 2 – 4 shows the data obtained from the experiment.

**TABLE 1:** Chemicals used in the study

Chemical name	Purity^a^	Source	CAS
DL-Proline	98%	Shanghai Macklin Biochemical Co., Ltd.	609-36-9
L-Serine	99%	Shanghai Macklin Biochemical Co., Ltd.	56-45-1
L-Glutamic acid	≧ 98.5%	Sinopharm Chemical Reagent Co., Ltd.	56-86-0
H_2_O	> 99.9%	Double-distilled water prepared by the laboratory	7732-18-5

^a^ The purity of the chemical is given by the producer.

**TABLE 2:** Experimental VLE data for temperature T, pressure P, and molality m for the DL-proline + H_2_O system^a^.

*m*_*1*_ = 11.460 mol/kg		*m*_*2*_ = 10.000 mol/kg		*m*_*3*_ = 8.000 mol/kg		*m*_*4*_ = 6.000 mol/kg		*m*_*5*_ = 4.000 mol/kg		*m*_*6*_ = 2.000 mol/kg	
*T/*K	*P/*kPa	*T/*K	*P/*kPa	*T/*K	*P/*kPa	*T/*K	*P/*kPa	*T/*K	*P/*kPa	*T/*K	*P/*kPa
318.15	6.55	313.65	5.25	316.45	7.06	315.55	6.9	311.95	5.3	312.05	4.7
328.05	10.95	325.65	10.25	324.75	10.72	325.75	11.9	321.55	9.9	324.65	11.9
335.85	15.65	336.65	17.75	333.75	16.12	333.05	17.3	330.55	16	329.65	15.5
341.05	20.45	341.15	22.25	340.45	23.12	337.55	21.4	335.85	20.8	335.65	21.2
346.15	25.65	345.45	26.75	343.25	26.32	342.55	27.1	340.15	25.4	339.45	25.9
350.15	30.05	349.15	31.25	349.05	33.82	345.75	31.4	343.85	30.1	343.75	30.1
355.45	37.65	352.55	36.75	351.95	37.92	349.85	37.2	347.55	35.3	346.95	35.8
357.05	40.15	355.05	40.75	354.65	42.72	352.25	41.1	351.45	41.5	350.45	41.4
360.55	46.15	359.85	48.25	357.15	47.31	356.45	48.4	354.15	46.3	353.05	45.9
363.25	51.45	362.45	52.55	359.55	51.81	358.45	51.9	356.15	50.2	355.25	50.4
366.05	57.15	364.65	57.25	362.55	56.8	360.85	56.5	358.95	55.8	358.05	56.4
367.95	61.15	366.45	61.25	364.95	62.3	362.85	60.9	361.05	60.3	360.25	60.9
371.95	69.45	371.85	72.95	368.95	70.3	367.55	71.5	366.15	70.8	364.45	70.1
376.15	80.65	374.85	81.25	372.55	80.3	371.05	80.5	368.95	79.4	367.95	80
378.85	89.15	377.35	88.55	375.35	88.8	374.45	90.4	372.65	90.8	370.75	88.4
382.75	101.15	381.25	101.25	379.05	101.3	377.45	101.4	375.75	100.8	374.55	100.9

^a^ Standard uncertainties u are u(P) = 0.01 kPa, u(T) = 0.05 K, and u(m) = 0.001 mol/kg (uncertainties in atmospheric pressure and temperature are caused by errors in the instrument itself).

**TABLE 3:** Experimental VLE data for temperature *T*, pressure *P*, and molality *m* for the L-serine + H_2_O system^a^.

m1 = 4.035 mol/kg		m2 = 3.000 mol/kg		m3 = 2.000 mol/kg		m4 = 1.000 mol/kg	
T/K	P/kPa	T/K	P/kPa	T/K	P/kPa	T/K	P/kPa
313.95	4.82	314.45	6.28	314.85	7.44	319.65	5.21
324.15	11.32	323.75	11.68	325.75	13.55	321.05	7.05
330.35	16	329.85	16.19	330.35	16.72	326.85	12.73
335.55	20.91	335.45	21.30	335.35	21.42	332.55	18.18
340.15	26.01	339.25	25.70	340.05	26.72	335.85	22.09
343.95	30.81	343.85	31.31	343.65	31.41	341.55	27.99
347.75	36.41	347.45	36.71	347.25	36.71	344.25	33.01
350.45	40.81	350.55	41.72	350.45	42.11	347.85	38.41
353.85	46.9	353.55	46.74	353.05	46.81	350.25	42.51
355.85	50.9	355.75	51.25	355.65	52.11	352.85	47.32
358.15	55.7	358.25	56.77	358.05	57.21	355.35	52.23
361.05	62.41	360.35	61.38	359.95	61.51	357.85	57.55
365.05	71.11	365.35	72.48	364.65	72.12	360.05	62.55
368.55	80.91	368.65	81.79	367.85	81.21	363.45	69.55
371.15	89.4			370.65	90.51	367.45	81.24
374.65	101.4			374.15	102.11	370.25	90.55
						373.85	102.58

^a^ Standard uncertainties u are u(P) = 0.01 kPa, u(T) = 0.05 K, and u(m) = 0.001 mol/kg (uncertainties in atmospheric pressure and temperature are caused by errors in the instrument itself).

**TABLE 4:** Experimental VLE data for temperature *T*, pressure *P*, and molality *m* for the L-glutamic acid + H_2_O system^a^.

*m*_*1*_ = 0.059 mol/kg		*m*_*2*_ = 0.050 mol/kg		*m*_*3*_ = 0.040 mol/kg		*m*_*4*_ = 0.030 mol/kg		*m*_*5*_ = 0.020 mol/kg		*m*_*6*_ = 0.010 mol/kg	
*T*/K	*P*/kPa	*T*/K	*P*/kPa	*T*/K	*P*/kPa	*T*/K	*P*/kPa	*T*/K	*P*/kPa	*T*/K	*P*/kPa
314.85	8	309.35	5.9	312.85	6.7	311.95	6.25	314.55	7.59	311.95	6.6
320.55	11	320.75	10.8	323.95	12.8	322.05	11.55	322.85	12.09	321.55	11.5
329.55	17.2	329.25	16.8	329.75	17.2	328.95	16.55	330.25	17.59	329.55	17.1
333.65	21	334.35	21.3	334.05	21.4	334.35	21.25	335.25	22.59	334.15	21.5
338.25	26.2	338.65	26.6	338.25	26	338.15	25.55	339.55	27.49	338.15	26.1
343.85	33.5	342.45	31.3	342.65	31.7	342.25	30.95	342.45	31.39	342.35	31.1
346.15	37	345.65	35.8	345.95	36.7	345.95	36.05	347.55	38.69	345.65	36.1
348.95	41.5	348.85	41	348.85	41.2	348.85	41.05	350.05	43.39	348.85	41.1
351.95	47	351.55	45.8	351.55	45.7	351.75	46.35	352.05	47.09	352.15	47.1
354.05	51.5	354.05	50.8	354.35	51.7	354.15	51.05	354.55	52.09	354.25	51.1
356.35	56.2	356.55	56.3	356.35	56	356.55	56.05	356.65	56.39	357.25	52.6
358.85	62.2	359.15	61	359.55	61.7	359.45	61.05	358.25	59.39	358.75	60.9
362.25	70.5	363.55	71.8	363.25	71.6	363.15	70.45	363.45	71.29	363.35	71.1
366.05	81	366.55	81.4	366.45	81.2	366.75	80.85	367.15	82.09	366.75	80.9
370.05	91.5	369.65	90.8	369.75	91.2	369.05	88.05	373.15	102.09	369.75	90.1
372.35	101.5	372.55	101.3	372.85	101.7	372.95	101.55			372.95	101.6

^a^ Standard uncertainties u are u(P) = 0.01 kPa, u(T) = 0.05 K, and u(m) = 0.001 mol/kg (uncertainties in atmospheric pressure and temperature are caused by errors in the instrument itself).

### 2.2 Apparatus

**FIGURE 2:**
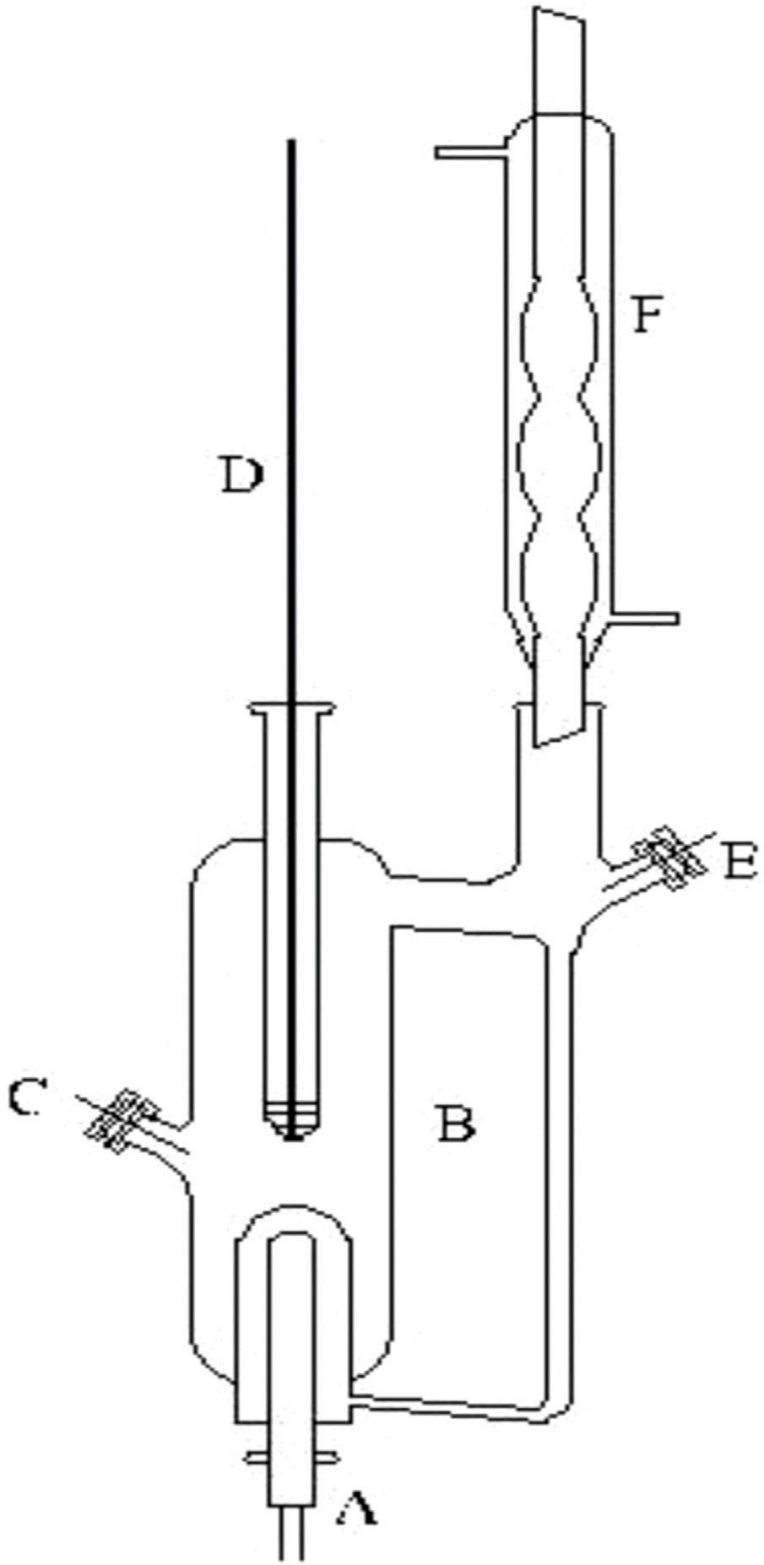
Schematic diagram of the VLE apparatus used in this work: **(A)** heating mantle, **(B)** equilibrium still, **(C)** sampling port, **(D)** thermometer well, **(E)** sampling port, and **(F)** condenser.

As shown in Figure 2, the measuring device for VLE of the dual circulation glass ebulliometer was analyzed as described previously (Xu et al., 2018). The device includes a vacuum pump, a pressure controller (model: Ruska Instrument Corp., Houston, United States), a heating mantle, and a temperature controller (model: SRS13A, SHIMADEN, Japan). In the present work, many relevant experimental details are described as follows: 1) when we added samples to the glass ebulliometer, the samples should be added to the proper position of the glass ebulliometer; this was to prevent too much or too little samples from causing experimental errors. 2) The ebulliometer was heated after adding the samples. 3) The judging standard of VLE is an important factor. The temperature on the temperature display remains the same for ∼2 min, and the condensate reflux of the ebulliometer is controlled at two to three drops per second and is stably refluxed for ∼2 min to establish an equilibrium state.

The infrared spectroscopy of three amino acids was analyzed using the Fourier transform infrared spectrometer at 298K. The instrument used was a Nicolet iS50 spectrometer equipped with a DTGS detector. Each spectrum was collected with the following parameters: 4 cm^−1^ resolution, 16 parallel scans, and a zero filling factor of 2. The ATR cell made of a trapezoidal diamond crystal was used.

### 2.3 Experimental procedure

#### 2.3.1 Sample preparation process

The required sample was weighed by using the analytical balance of model: BSA2245, dissolved in water, and completely dissolved by ultrasonic-assisted dissolution. The ultrasonic-assisted dissolution (model: SK3210LHC) conditions were 53 Hz, 28°C, and 50% power.

#### 2.3.2 Experimental process

During the experiment, the pressure was controlled by the pressure control valve, vacuum pump, and pressure sensor, and the heating temperature was controlled by the temperature controller. The VLE of the amino acid aqueous solution was measured at pressures between 4.7 and 102.58 kPa. The maximum pressure measured by the VLE experiment was 102.58 kPa due to the daily variation in atmospheric pressure, while the maximum value of the atmospheric pressure was 102.58 kPa throughout the experimental period. The specific experimental procedure was described as follows: 1) first, the air tightness of the instrument must be checked. 2) Temperature and pressure detectors were calibrated. .3) The sample (40 ml) prepared was poured above into the dual circulation glass ebulliometer. Before loading the sample, the ebulliometer does not need to be purged with a gas, just add the sample directly. 4) The heating switch was turned on (the voltage was in the range of 100–150 kV), and the vacuum pump was turned on. 5) The pressure inside the container was changed through the pressure valve. 6) The equilibration time for the experiment was 5–10 min. 7) When VLE equilibrium was reached, we recorded the temperature and pressure values. During the experiment, there were two standard methods for us to judge whether the VLE was reached. One was to observe the temperature variation on the temperature display, and the other was to observe the reflux speed of the condensed water in the condenser tube. The specific judgment method is discussed in Section 2.2.

### 2.4 Quantum chemical calculations

The Gaussian 16 package was employed to perform the quantum chemical calculations (Frisch et al., 2016). To optimize the geometries, vibrational frequencies, and energies of the isolated single molecules (DL-proline, L-serine, L-glutamic acid, and H_2_O), the three amino acid dimers, and their complexes with H_2_O, the M06-2X method was used with the 6–311++G** basis set (James et al., 2011, Walker et al., 2013, Holland et al., 2016). All the optimized geometries were confirmed to be local minima with no imaginary frequencies. The interaction energy was estimated as the difference between the total energy of a complex and those of the corresponding minimum energy monomers.

## 3 Model description

The model for the excess Gibbs energy is expressed by the NRTL term (Xu et al., 2018):

$$\frac{n_{t}G_{NRTL}^{e}}{RT}=m_{x}m_{w}\left(\frac{\tau_{w,x}G_{w,x}}{m_{x}+m_{w}G_{w,x}}+\frac{\tau_{x,w}G_{x,w}}{m_{w}+m_{x}G_{x,w}}\right)$$ (1)

$$G_{w,x}=\exp\left(-\alpha \tau_{w,x}\right)$$ (2)

$$G_{x,w}=\exp\left(-\alpha \tau_{x,w}\right)$$ (3)

where n_t_ is the molar of the solute and solvent and m_x_ is the total molality of the solute, *α* = 0.3. Since the NRTL equation contains three parameters, a large number of binary system experimental data collations show that α varies from about 0.20 to 0.47. In the absence of experimental data, the value of α can often be arbitrarily specified, and a typical choice is *α* = 0.3 (Prausnitz et al., 1999).

The reference state of activity coefficients in the excess Gibbs energy model is γ_i_→1 as x_i_ (=n_i_/n_t_)→1.

It is assumed that the solute in the electrolyte solution and *h* water molecules exists as an entity molecule in this model. Hydrated entity molecules and free water molecules exist in the solution:

$$m_{w}=\frac{1000}{Ms}-\overset{n}{\underset{i=1}{\sum }}\left(h_{i}m_{i}\right)$$ (4)

where h_i_ is the hydration numbers of the solute, n is the number of species of the solute in an electrolyte solution, Ms is the molecular weight of water, m_i_ is the molality of the solute, and m_w_ is the molal of free water.

In this work, all the solutes in the mixed electrolyte solution are assumed to be an entity; therefore, the solution is assumed to have only “one solute entity” and solvent, so we assume that the mixed electrolyte solution is a binary solution (Xu et al., 2014).

τ_w,x,_ and τ_x,w_ are the water-entity term and entity-water term, respectively:

$$\tau_{w,x}=\overset{n}{\underset{i=1}{\sum }}\left(\tau_{w,i}m_{i}\right)/\overset{n}{\underset{i=1}{\sum }}\left(m_{i}\right)$$ (5)

$$\tau_{x,w}=\overset{n}{\underset{i=1}{\sum }}\left(\tau_{i,w}m_{i}\right)/\overset{n}{\underset{i=1}{\sum }}\left(m_{i}\right)$$ (6)

where τ_w,i_ and τ_i,w_ are the water–solute parameter and solute–water parameter, respectively.

$$RT\ln\gamma_{i}=\left(\frac{\partial n_{t}G_{NRTL}^{e}}{\partial n_{i}}\right).$$ (7)

The Gibbs–Duhem equation is described as follows:

$$\underset{i}{\sum }x_{i}d\ln\gamma_{i}=0$$ (8)

Through the derivation of the aforementioned thermodynamic equation, we obtain the activity of water from Eqs 1, 7, and 8, and the final equation can be written as follows:

$$\ln a_{w}=\left(\frac{\overset{n}{\underset{i=1}{\sum }}\left(\tau_{w,i}m_{i}\right)G_{w,x}}{\overset{n}{\underset{i=1}{\sum }}\left(m_{i}\right)+m_{w}G_{w,x}}+\frac{\overset{n}{\underset{i=1}{\sum }}\left(\tau_{i,w}m_{i}\right)G_{x,w}}{m_{w}+\overset{n}{\underset{i=1}{\sum }}\left(m_{i}\right)G_{x,w}}\right)+m_{w}\left(\frac{-\overset{n}{\underset{i=1}{\sum }}\left(\tau_{w,i}m_{i}\right)G_{w,x}^{2}}{{\left(\overset{n}{\underset{i=1}{\sum }}\left(m_{i}\right)+m_{w}G_{w,x}\right)}^{2}}-\frac{\overset{n}{\underset{i=1}{\sum }}\left(\tau_{i,w}m_{i}\right)G_{x,w}}{{\left(m_{w}+\overset{n}{\underset{i=1}{\sum }}\left(m_{i}\right)G_{w,x}\right)}^{2}}\right)+\ln\left(\frac{1000/Ms}{1000/Ms+\overset{n}{\underset{i=1}{\sum }}\left(m_{i}\right)}\right)$$ (9)

Water activity between 298.15 and 382.75 K was tested with this method. To correlate data at different temperatures, the following temperature dependence of the parameters τ_w,i_ and τ_i,w_ is used:

$$\tau_{i,w}=\tau_{i,w}^{\left(0\right)}+\tau_{i,w}^{\left(1\right)}/T$$ (10)

$$\tau_{w,i}=\tau_{w,i}^{\left(0\right)}+\tau_{w,i}^{\left(1\right)}/T$$ (11)

In the final model, five parameters (h, $\tau_{w,i}^{\left(0\right)}$ , $\tau_{w,i}^{\left(1\right)}$ , $\tau_{i,w}^{\left(0\right)}$ , and $\tau_{i,w}^{\left(1\right)}$ ) were fitted to our experimental data.

After the thermodynamic derivation of the excess Gibbs energy, Eq. 9 can be obtained, which is the final thermodynamic model formula and can be directly used to correlate and fit the VLE data. This equation directly uses the data on the vapor–liquid equilibrium (activity) of the amino acid aqueous solution to correlate the parameters (five parameters) in fitted Eq. 9. Finally, the parameters obtained by correlation fitting are directly substituted into the thermodynamic model to calculate the equilibrium data, and the feasibility of the thermodynamic model is verified by comparing the calculation results with the experimental results.

## 4 Results and discussion

### 4.1 Discussion of amino acid aqueous solution solubility

In this article, the feasibility of the device was demonstrated in the previous work, and the accuracy of the experimental results was verified (Xu et al., 2018).

**FIGURE 3:**
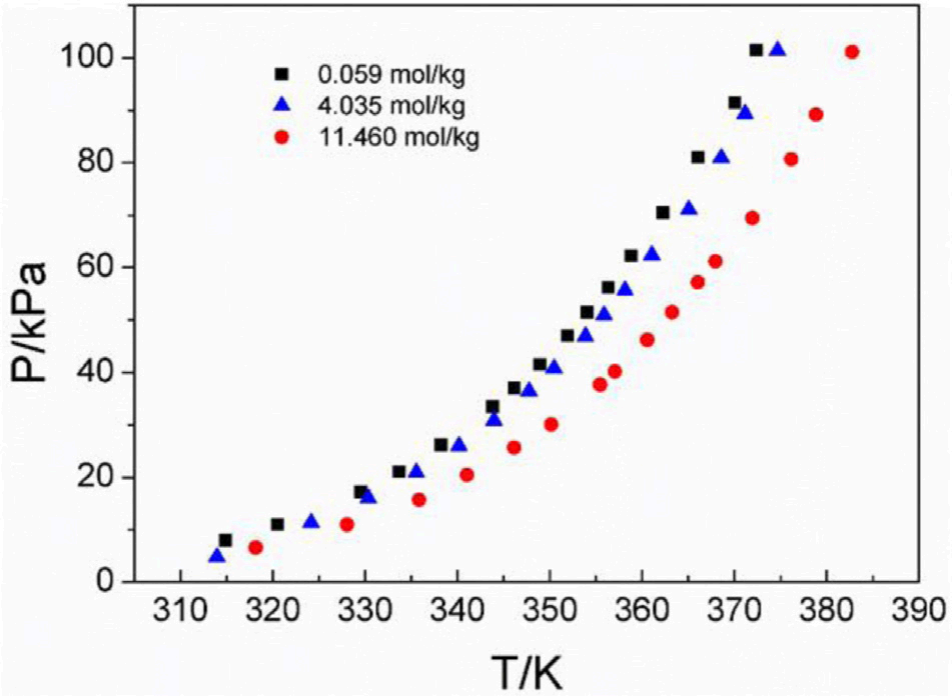
Vapor–liquid equilibrium in the three different saturated amino acid aqueous solutions. Full symbols represent experimental data (this work). Black square represents L-glutamic acid, blue triangle represents L-serine, and red circle represents DL-proline.

Three amino acids with great differences in solubility were selected as follows: L-glutamic acid solubility in aqueous solutions is 0.059 mol/kg, L-serine solubility in aqueous solutions is 4.04 mol/kg, and DL-proline solubility in aqueous solutions is 11.46 mol/kg at atmospheric pressure and room temperature (25°C) (Jin et al., 1992; Dalton et al., 1933; Fasman et al., 1976). Figure 3 shows the P-T-m diagram of three different saturated amino acid aqueous solutions.

As shown in Figure 3, the saturated vapor pressures of the three different amino acid aqueous solutions in the saturated state are also different due to the different solubilities at the same temperature. In addition, it can be seen that with the increase in temperature, the saturated vapor pressure of amino acids increases continuously, which is consistent with the description in the literature (Ding et al., 2021).This could be caused by the same temperature. For the same volume of solution sample, the number of solvent molecules in the unit volume and unit surface of the sample with greater solubility decreases. Therefore, the number of solvent molecules that may leave the liquid surface and enter the gas phase decreases per unit time. Thus, the solvent and its vapor can reach equilibrium at a lower vapor pressure. This is also consistent with the experimental results at the same temperature. The saturated vapor pressure of the most soluble DL-proline is the lowest, and the saturated vapor pressure of the least soluble L-glutamic acid is the highest. In addition, it also corresponds to the infrared spectrum of the amino acid aqueous solutions described later. With the increasing concentration of amino acid aqueous solutions, the characteristic peaks of *v* (C=O) move likely to lower wavenumbers, and the hydrogen bonds involving the amino acid *v* (C=O) are red-shifted. This means that the intermolecular interactions increase. Combined with the law of the VLE plots, in a high concentration or high solubility system, the intermolecular interactions are large, and the saturated vapor pressure is low.

### 4.2 Discussion of experimental results and correlation of models

**FIGURE 4:**
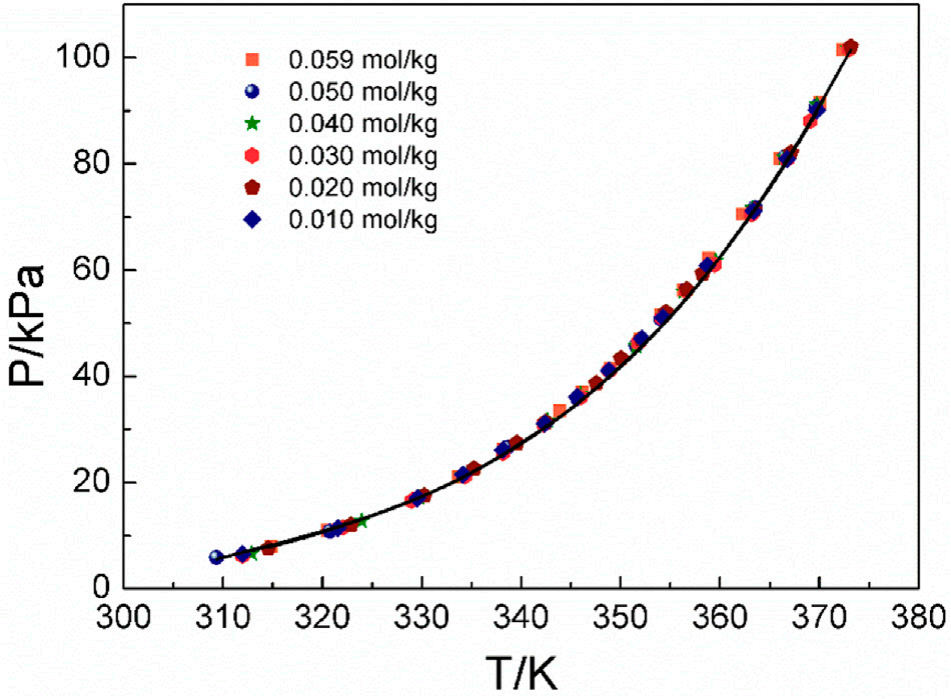
Correlation of experimental VLE data for the L-glutamic acid aqueous solution. Symbols represent experimental data (this work), and lines represent the correlation of the model.

**FIGURE 5:**
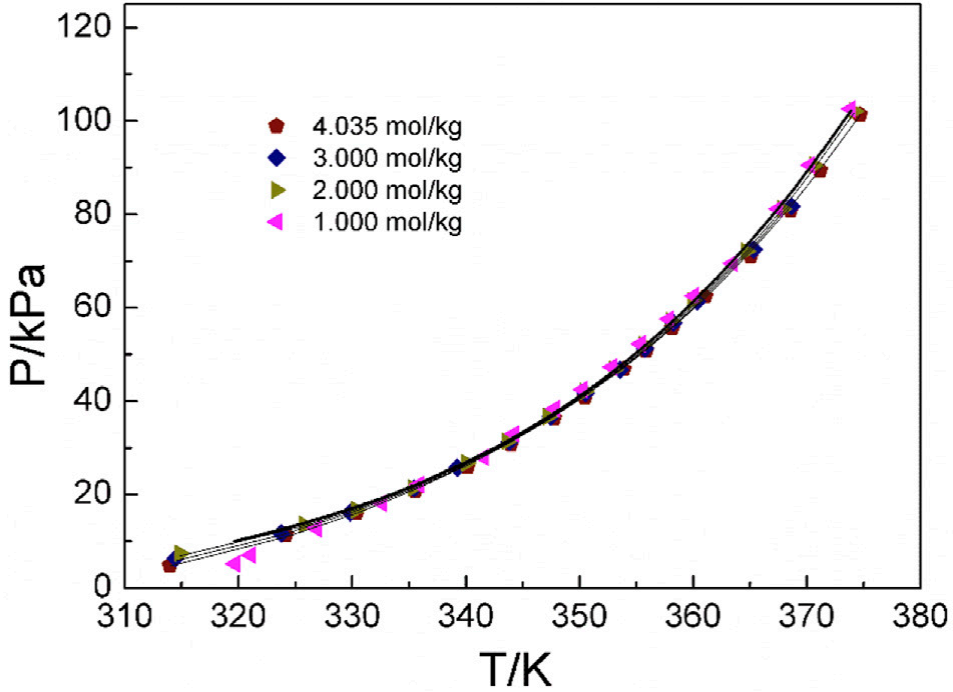
Correlation of experimental VLE data for the L-serine aqueous solution. Symbols represent experimental data (this work), and lines represent the correlation of the model.

**FIGURE 6:**
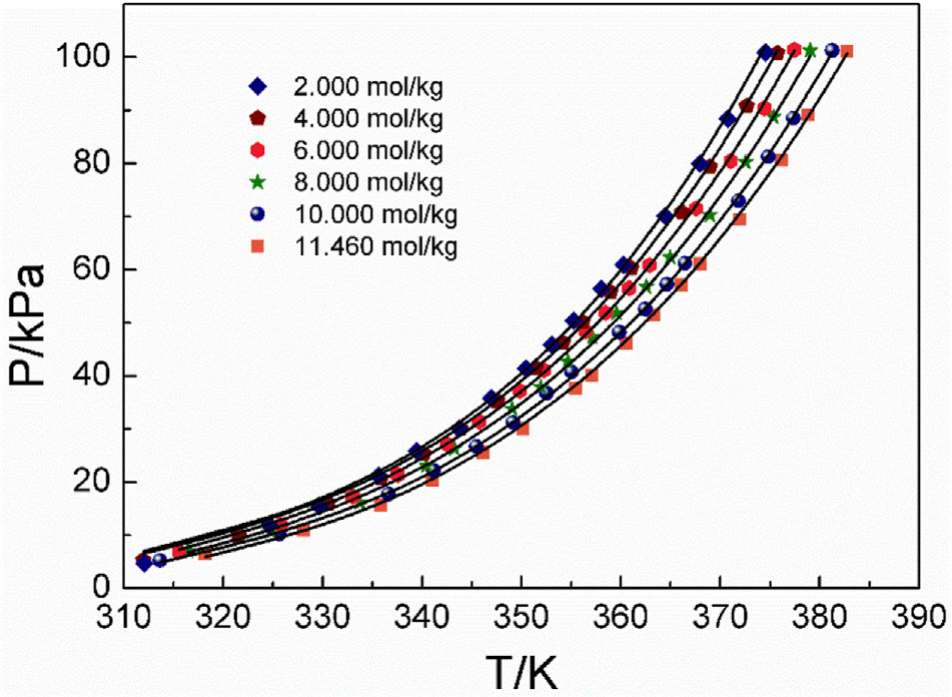
Correlation of experimental VLE data for the DL-proline aqueous solution. Symbols represent experimental data (this work), and lines represent the correlation of the model.

Figures 4–6 display the P-T-m plots of different kinds and concentrations of amino acids in aqueous solutions. Figure 4 shows that the saturated vapor pressure increases with the increasing temperature, and different amino acids show the same changes even if they present different solubilities (DL-proline, 11.46 mol/kg; L-serine, 4.04 mol/kg; and L-glutamic acid, 0.059 mol/kg). In this work, three amino acids were investigated, which are DL-proline, L-serine, and L-glutamic acid aqueous solutions, and their solubility is 11.46, 4.04, and 0.059 mol/kg, respectively. Figures 5, 6 present that for the same amino acid aqueous solutions, the saturated vapor pressure of amino acid aqueous solutions with high concentrations was low at the same temperature. However, due to the low solubility of L-serine and L-glutamic acid aqueous solutions, within the solubility range, the P-T-m plots did not change significantly when the concentration was changed.

Moreover, the NRTL-X model (Xu et al., 2019) was applied to correlate the VLE results, as illustrated in Figures 4–6. The correlation procedure is as follows: in this part, 1stOpt 9.0 was chosen as the main calculation tool. 1stOpt 9.0 was used to model the VLE data. The model described previously is strictly a semi-empirical model. The hydration hypotheses and the model have been proposed in the previous work (Xu et al., 2019). The interaction term is remodeled based on 1stOpt 9.0 calculation data. The model is described in Eqs 1–11. The model parameters for the three different amino acids are listed in Table 5. It could be concluded from Figures 3–5 that the experimental values are in good agreement with the model values, which indicates a good correlation of the experimental data with this model. The results show that for three amino acid aqueous solutions, dY = 0.27 kPa and the average of dP = 0.43%. dY and dP are calculated as follows:

$$dY=\left(1/N\right)\sum \left|P_{\exp}-P_{cal}\right|$$ (12)

$$dP=\left(1/N\right)\sum \left|P_{\exp}-P_{cal}\right|/P_{\exp}\times 100\%$$ (13)

where N is the number of data points, P_exp_ represents the experimental pressure, and P_cal_ represents the calculated pressure.

**TABLE 5:** Model parameters for binary electrolyte solutions in the model.

System	h	$\tau_{w,i}^{\left(0\right)}$	$\tau_{w,i}^{\left(1\right)}$	$\tau_{i.w}^{\left(0\right)}$	$\tau_{i,w}^{\left(1\right)}$
L-Glutamate	0.2	113	17.43	−39447.30	2594.06
L-Serine	0.25	41.19	−31.97	−14810.23	12616.58
DL-Proline	0.3	2.8	−27.16	−2111.82	11534.73

### 4.3 Infrared spectrum analysis of amino acid aqueous solutions

**FIGURE 7:**
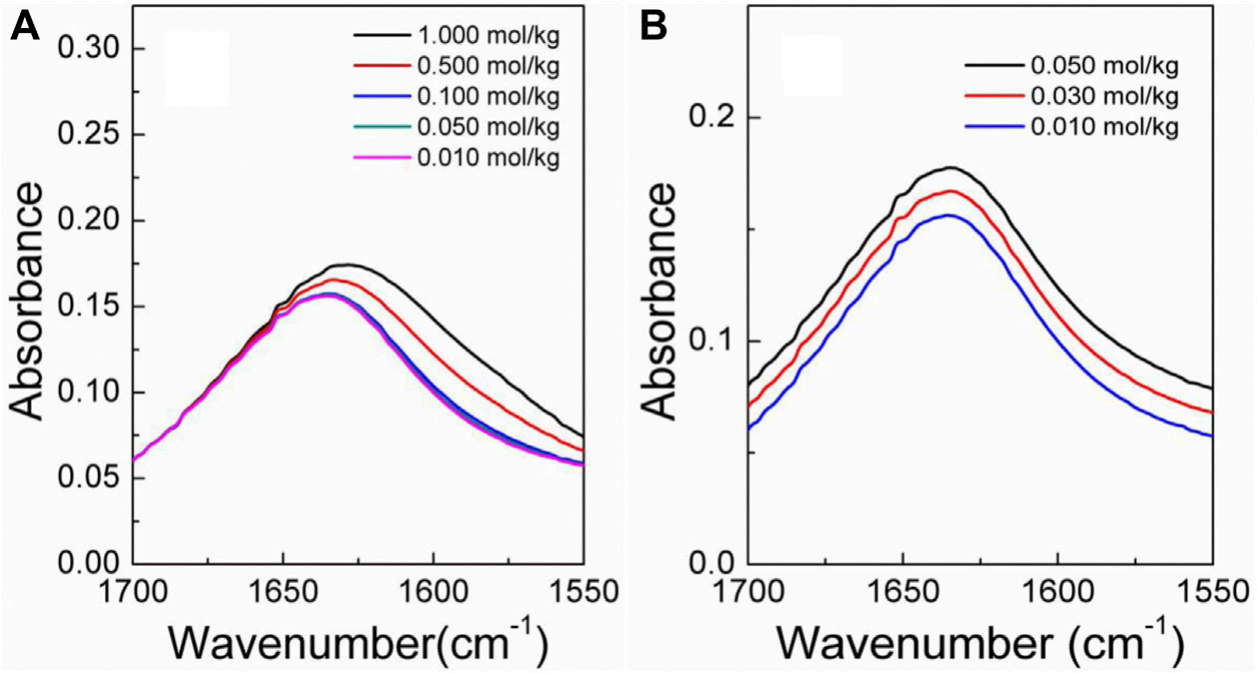
Infrared spectra of the DL-proline aqueous solution in the range of *v* (C=O). **(A)** Actual curve. **(B)** Elevated curve of **(A)**.

**FIGURE 8:**
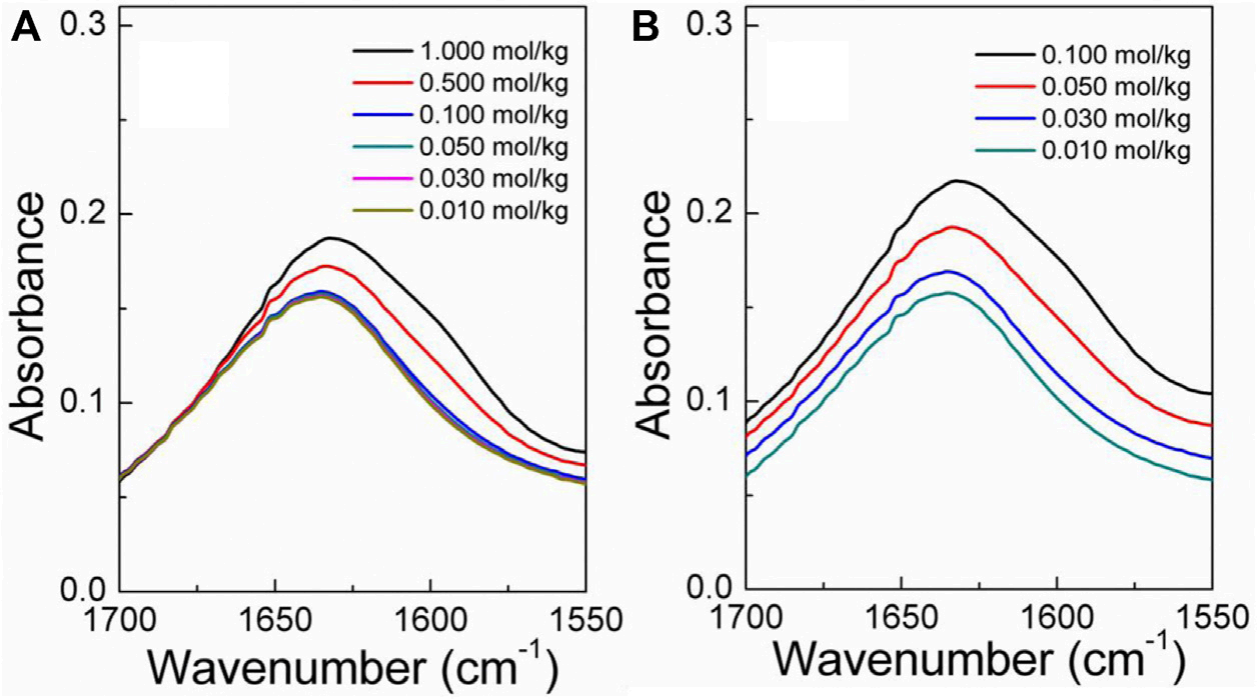
Infrared spectra of the L-serine aqueous solution in the range of v (C=O). **(A)** Actual curve. **(B)** Elevated curve of **(A)**.

**FIGURE 9:**
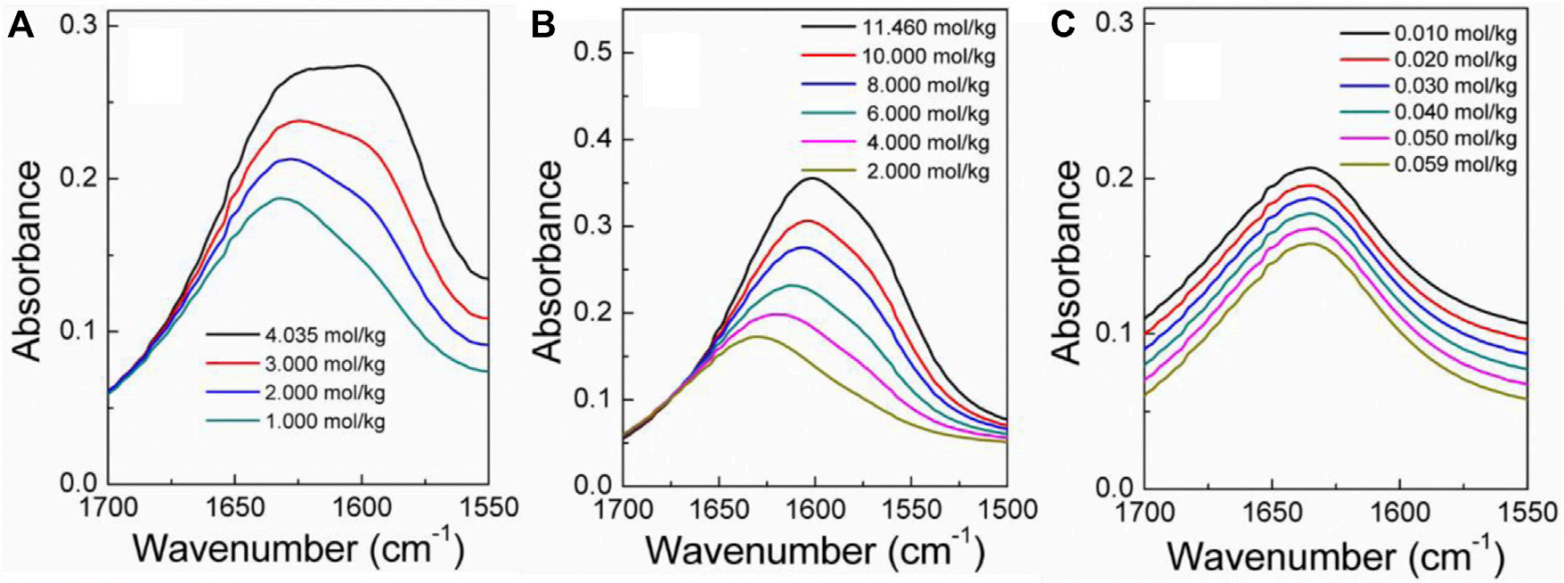
Infrared spectra **(A–C)** of L-serine, DL-proline, and L-glutamic acid aqueous solutions in the range of *v* (C=O). **(A,B)** Actual curve. **(C)** Elevated curve.

The infrared spectra of the amino acid aqueous solutions are shown in Figures 7–9. The intermolecular interactions of amino acid aqueous solutions are analyzed from the perspective of infrared spectroscopy. Figure 9 shows the absorption peak of ν(C=O) at approximately 1600 cm^−1^ of amino acid aqueous solutions. However, this phenomenon was evident in DL-proline and L-serine aqueous solutions, but not in L-glutamic acid aqueous solutions, which may be due to the low solubility of L-glutamic acid and the low concentration of the prepared samples. Therefore, to verify the accuracy, infrared spectra measurements were also performed on samples with low concentrations of L-serine and DL-proline aqueous solutions, and the results are reported in Figures 7, 8. ν (C=O) in L-serine and DL-proline aqueous solutions in low concentration showed a mild red-shift. The literature showed that the redshift of the peak position of ν (C=O) indicates the strengthening of hydrogen bonding interactions (Zhou et al., 2018). In addition, quantum chemical calculations were used to further demonstrate this point. Gaussian 16 software with the M06-2X/6–311++G** method was used to calculate the geometries and vibrational frequencies.

**FIGURE 10:**
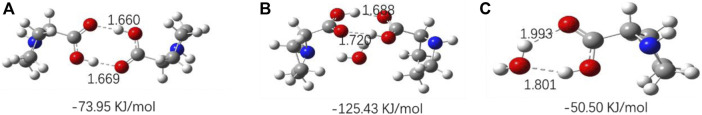
Optimized geometries for the amino acid complexes. **(A)** DL-Proline (dimer); **(B)** DL-proline + H_2_O complexes; and **(C)** DL-proline–H_2_O complexes; hydrogen bonds are denoted by dashed lines, and the corresponding H···O and N···H distances are labeled. The interaction energy of each complex is noted below the structure.

**FIGURE 11:**
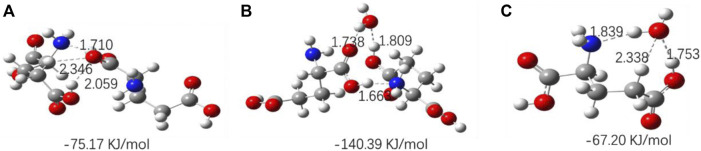
Optimized geometries for the amino acid complexes. **(A)** L-Glutamic acid (dimer); **(B)** L-glutamic acid–H_2_O complexes; **(C)** L-glutamic acid + H_2_O complexes; hydrogen bonds are denoted by dashed lines, and the corresponding H···O and N···H distances are labeled. The interaction energy of each complex is noted below the structure.

**FIGURE 12:**
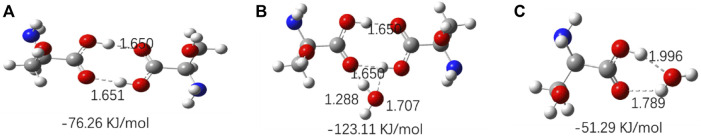
Optimized geometries for the amino acid complexes. **(A)** L-Serine (dimer); **(B)** L-serine–H_2_O complexes; **(C)** L-serine + H_2_O complexes; hydrogen bonds are denoted by dashed lines, and the corresponding H···O and N···H distances are labeled. The interaction energy of each complex is noted below the structure.

The optimized geometries of three amino acids’ monomer, dimer, and their complexes with H_2_O are shown in Figures 10–12, and their corresponding calculated frequencies are shown in Table 6. As shown in the figure, all three amino acids can form hydrogen bonds with H_2_O. In Table 6, compared with amino acid molecules, the calculated frequencies of *v* (C=O) in amino acid−water complexes show redshift. The results indicate that redshift means the formation or enhancing hydrogen bonds of *v* (C=O), which is consistent with the results in infrared spectra. In addition, it can be seen from Figures 10–12 that the three amino acid dimers can form strong hydrogen bonding interactions. In addition, water can form a stronger hydrogen bond with amino acid dimers than amino acid monomers. All those result support that hydrogen bonding interactions are stronger in high concentrations of amino acid aqueous solutions. In addition, DL-proline and L-serine dimers all form stable planar double hydrogen bonds. The two amino acids are more difficult to aggregate into clusters than L-glutamic acid, which is consistent with the experimental result that the L-glutamic acid has the lowest solubility.

**TABLE 6:** Calculated frequencies of *v*(C=O) (cm^−1^) for the amino acids, amino acids with water complexes, and amino acid dimer complexes.

	Frequency		Frequency		Frequency		Frequency
DL-Proline	1876.92	DL-Proline−H_2_O	1831.16	2DL-Proline	1815.42	2DL-Proline−H_2_O	1822.35
L-Glutamic acid	1867.75	L-Glutamic acid−H_2_O	1820.53	2L-Glutamic acid	1890.54	2L-Glutamic acid−H_2_O	1875.78
L-Serine	1867.71	L-Serine−H_2_O	1820.12	2L-Serine	1806.99	2L-Serine−H_2_O	1796.85

## 5 Conclusion

The VLE for the amino acid aqueous solutions was obtained by using a VLE dual circulation glass ebulliometer. Under the pressure of 4.82–102.58 kPa, the experimental data were measured. The P-T-m diagrams were obtained, revealing that the saturated vapor pressure increases at a certain rate as the temperature increases. In addition, the diagram also depicts the magnitude of the difference in VLE between the different samples. Furthermore, an improved NRTL-X model was proposed to correlate the VLE experimental data. This model was derived from the excess Gibbs energy model. In addition, in this work, the dY and dP values used by the NRTL-X model were small, and there were few thermodynamic models for amino acid aqueous solutions. The results showed that the calculated values of the model fit well with the experimental data, so this model was considered valuable.

Combining the P-T-m diagram, the infrared spectrum, and the quantum chemical calculation, it could be concluded that at the same temperature, such as 25°C, the vapor pressure of the same amino acid decreases with the increase in the concentration, and the redshift of ν (C=O) increases with the increase in amino acid concentration in the infrared spectrum. Therefore, the principle of different solubilities of amino acid aqueous solutions and the change of infrared spectrum wavenumber of amino acid aqueous solution with different concentrations are explained from the microscopic point of view. It lays the foundation for the study of other properties of amino acids (Renon and Prausnitz, 1968; Abrams and Prausnitz, 1975; Jia et al., 2020; He et al., 2021; Arnautovic et al., 2022).

## Data Availability

The original contributions presented in the study are included in the article/Supplementary Material; further inquiries can be directed to the corresponding authors.

## Nomenclature

α: activity
G_e_: excess Gibbs energy, J•mol^-1^
R: gas constant, J•mol^-1^•kg^-1^
m: molality, mol•kg^-1^
n: mole, mol
m_x_: total molality of solute, mol•kg^-1^
m_w_: molar of free water, mol•kg^-1^
*h*: hydration numbers of the solute
Z: solvation parameters
T: temperature, K
M_s_: molecular weight of water
γ: activity coefficients
n_t_: integral molar quantity, mol
τ: parameter
dY: mean absolute error, mol•kg^-1^
dP: mean relative error, %
